# Prognostic Value of Lymph Node Ratio (LNR) in Patients with Postoperative N2 Feature in Non-Small Cell Lung Cancer (NSCLC)

**DOI:** 10.3390/jcm13154570

**Published:** 2024-08-05

**Authors:** Mariusz Piotr Łochowski, Justyna Chałubińska-Fendler, Aleksandra Szlachcińska, Barbara Łochowska, Daniel Brzeziński, Jacek Kaczmarski, Józef Kozak

**Affiliations:** 1 Department of Thoracic Surgery and Respiratory Rehabilitation, Medical University of Lodz, Regional Multi-Specialist Center for Oncology and Traumatology, Nicolaus Copernicus Memorial Hospital, 93-513 Lodz, Poland; witaszczyk@hotmail.com (A.S.); daniel.b@autograf.pl (D.B.); kaczmarski1970@gmail.com (J.K.); thorsurg@wp.pl (J.K.); 2 Department of Radiotherapy, Military Institute of Medicine—National Research Inisitute, 04-141 Warsaw, Poland; jchalubinska-fendler@wim.mil.pl; 3 Department of Radiotherapy and General Oncology, Regional Multi-Specialist Center for Oncology and Traumatology, Nicolaus Copernicus Memorial Hospital, 93-513 Lodz, Poland; blochowska@op.pl

**Keywords:** non-small cell lung cancer, N2 stage, LNR ratio

## Abstract

**Introduction:** One of the most important prognostic factors in non-small cell lung cancer (NSCLC), a condition with a high mortality rate, is the presence of mediastinal lymph node metastases alongside distant metastases. The aim of this study was to evaluate the prognostic value of selected parameters of N2 stage NSCLC with a special focus on lymph node ratio (LNR). **Material:** The study included 163 patients (61 women and 102 men) operated on due to NSCLC, postoperatively diagnosed as stage N2. The age of the patients ranged from 38 to 82 years (mean age: 62.4 years). The effects of the following factors on clinical data and survival rate were assessed: N1 stage, total number of all metastatic nodes, LNR and LNR N2 ratios, and the presence of skip, single- or multistation metastases. **Results:** Univariate analysis showed patient survival to be correlated with LNR and LNR N2 ratios, single/multistation metastases, and the number of nodes involved in metastasis. A multivariate model based on patient clinical data found nicotine dependence (*p* = 0.013), LNR > 0.26 (*p* = 0.004), and Charlson Comorbidity Index (CCI) value > 3 (*p* = 0.014) to be independent adverse prognostic factors in this group. **Conclusions:** LNR ratio is a significant cancer disease-derived independent prognostic factor for patients with postoperative N2 stage NSCLC. In addition, smoking and comorbidities also appear to have prognostic value.

## 1. Introduction

Despite advances in the diagnostics and treatment of oncological diseases, a significant challenge is still posed by lung cancer, which is responsible for the largest number of deaths worldwide. It was estimated that more than 1,918,000 new cases of cancer would be detected in the United States in 2022, and these will result in 610,000 deaths. One particularly prevalent form is lung cancer, of which 350 people are estimated to die each day [1]. The most important prognostic factor is considered to be tumor stage, indicated by the presence of mediastinal lymph node metastases and distant metastases [2]. 

Patients with mediastinal lymph node metastases (N2 stage) are classified as TNM stage IIIA–IIIB. The 8th edition of the TNM classification introduced major changes to T and M descriptors [3]. None of the previous editions of the TNM classification of lung cancer included N2 node stage parameters such as the volume of nodes, involved station, number of lymph nodes, or presence of skip metastases. The number of involved lymph nodes is represented by the lymph node ratio (LNR). However, while LNR has been found to have prognostic value in pancreatic, breast, and laryngeal cancer [4], previous studies have obtained uneven results, and its value in lung cancer remains unclear, mainly due to the variable numbers of lymph nodes collected intraoperatively [5].

Furthermore, in the era of rapid development of minimally invasive techniques, the subject of this study gets more complicated due to inconsistent opinions regarding the quality, scope, and technique of lymphadenectomy [6].

The aim of the present study is to determine the prognostic value of LNR and LNR-N2 ratios, and some other features of metastatic N2 nodes, in patients undergoing open surgery for non-small cell lung cancer (NSCLC). 

## 2. Material

### 2.1. Study Population

Between 2007 and 2017, 1285 patients with lung cancer were treated surgically at the Department of Thoracic Surgery in Nicolaus Copernicus Hospital in Lodz. This group included 163 patients with preoperative stage IA–IIB, as well as those at stage IIIA (i.e., involvement of a single N2 node group which could be surgically removed indicated by PET CT), and some with N2 stage determined postoperatively. The exclusion criteria included patents diagnosed with small cell lung cancer or carcinoid tumors, or patients who underwent wedge resection due to low FEV1 values. 

The study was approved by the Bioethics Committee of the Medical University of Lodz (no. RNN/83/19/KE). It was based on the principles of the Declaration of Helsinki (updated in 2013). All patient personal data were considered confidential. Due to the retrospective nature of the study, no Bioethics Committee consent was required to access the medical records of individual patients.

### 2.2. Patient Characteristics

The group comprised 163 patients (61 women and 102 men), aged 38 to 82 years (mean age: 62.4 years). Of the group, 61% admitted nicotine dependence. 

The indication for surgery was the presence of NSCLC, located in the right lung in 76 patients and in the left lung in 87 patients. Preoperative histopathological examination revealed squamous cell carcinoma in 70 cases, adenocarcinoma in 78 cases, multicellular carcinoma in 11 cases (including two neuroendocrine forms), and mixed forms (adenosquamous) in 4 cases. Almost half of the patients (80 patients) scored three points according to the Charlson Comorbidity Index (CCI), 74 received two points, and another 74 received four points (Table 1).

### 2.3. Surgical Treatment, Perioperative Complications, Adjuvant Therapy

All lobectomies, bilobectomies, and pneumonectomies were performed using a standard technique, though anterolateral thoracotomy was carried out under general anesthesia with the use of a double-lumen tube. In patients with cancer located in the right lung, lymph nodes from groups 2R, 3A, 4R, 7, 8, 9, 10, and 11 (according to the Japan Lung Cancer Society classification [7]) were removed. In the patients with cancer of the left lung, groups 3A, 4L, 5, 6, 7, 8, 9, 10, and 11 were removed. At least six lymph node stations of N1 and N2 stage were removed en bloc: the minimum number of dissected lymph nodes was six. 

Postoperative lung cancer staging was assessed according to the TNM classification (UICC 2017—8th edition) [8], and the removed lymph nodes were classified according to the Naruke map [9]. All patients receiving surgical treatment underwent R0 resection. Lobectomy was performed in 102 patients (63%), pneumonectomy in 43 (26%), and bilobectomy in 18 (11%). Based on postoperative histopathological examination, 111 (68%) patients were categorized into stage III A and 52 (32%) patients into stage III B. 

Two patients died in the postoperative period: one due to ischemic stroke, the other one due to respiratory failure. Another two patients died within 30 days following surgery. Complications were noted in 17 patients (10%): prolonged air leakage was observed in five cases, lung atelectasis requiring bronchoscopy in four, and postoperative blood transfusion was necessary in three. Atrial fibrillation and bronchial fistula were reported in the remaining patients (Table 2).

In total, 141 (86%) patients received adjuvant treatment, mostly based on cisplatin. Ten patients did not give their consent for adjuvant treatment. Chemotherapy was discontinued in eight patients due to age and multiple comorbidities. Unfortunately, it was not possible to collect accurate molecular data regarding the treatment, as it was provided outside the medical center. 

### 2.4. Statistical Methods

The prognostic values of N1 stage, total metastatic node number, LNR and LNR-N2 ratios, and the presence of skip, single- or multistation metastases were determined by statistical analysis. The analysis included the following clinical data: age, sex, nicotine dependence, histopathological diagnoses, degree of severity, TNM classification, CCI index, extent of surgery, and patient survival. LNR was calculated as the ratio of metastatic lymph nodes to all lymph nodes removed during surgery. LNR-N2 was defined as the ratio of the number of N2-stage metastatic lymph nodes to the number of all dissected lymph nodes (it was our innovation). 

Continuous variables were compared with the Shapiro–Wilk test. As the parameters were not normally distributed, the values were presented as medians and interquartile range (25–75%), and further analyses were performed using non-parametric tests.

Pairs of independent continuous variables were compared with the Mann–Whitney U test, while sets of more than two groups were tested with the Kruskal–Wallis test with the Dunn–Bonferroni post hoc test. Nominal variables, i.e., number of observations and percentage values, were calculated for the study and control groups, and compared using the Chi^2^ test. Survival analysis was performed using the Kaplan–Meyer method. Nominal parameters were subjected to univariate analysis using the log-rank test. Continuous variables were subjected to univariate and multivariate analysis using the Cox proportional hazard test (Cox-PHR).

The maximum sensitivity and specificity of the CCI index were determined by AUC (area under the curve) analysis based on Receiver Operating Characteristic (ROC) curves. The cut-off points for continuous variables found to be significant by univariate survival analysis were determined using the AUC values based on the Youden method. The separation of survival curves was drawn using the calculated cut-off point. 

All variables were used to construct a Cox-PHR model. All variables with a *p*-value below 0.2 in the Cox hazard model were then excluded when constructing the final model.

## 3. Results

### 3.1. Postoperative Survival 

**Figure 1:**
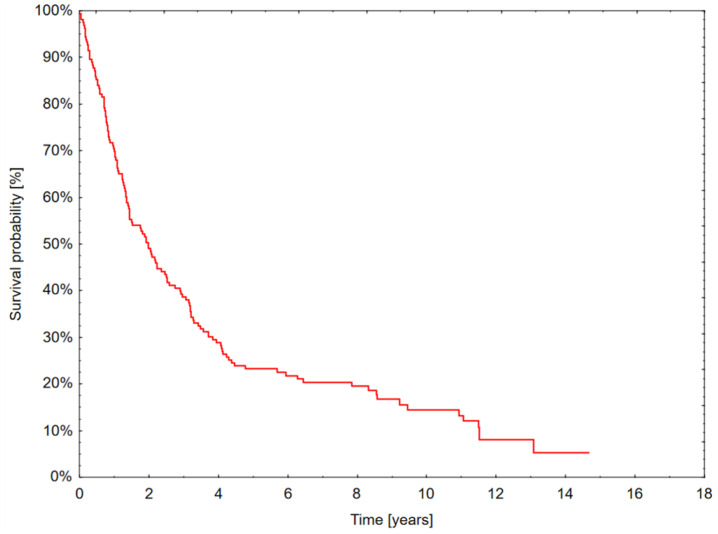
Overall survival curve of the patients.

The median patient survival was 1.95 years. Of the studied patients, 69.9% survived one year, 48.8% two years, and 38.3% three years; five-year survival was achieved by only 23.1% (Figure 1).

Survival was not significantly influenced by patient age (*p* = 0.213) or sex (*p* = 0.49). While longer survival was noted for patients after bilobectomy (3.17 years) compared to those after lobectomy (1.88 years) or pneumonectomy (2.11 years), this difference was statistically insignificant (*p* = 0.69). Patients with CCI > 3 demonstrated a significantly shorter survival time (2.49 years) compared to those with CCI 3 or lower (1.42 years; *p* = 0.019). A cut-off value of three nodes was determined by the Youden method: AUC value 0.302 (95% CI: 0.072–0.533, *p* = 0.01). Survival was not significantly influenced by tumor stage (IIIA versus III B), location of the primary lesion, or type of histopathological diagnosis (*p* = 0.1 *p* = 0.68 and *p* = 0.07, respectively).

### 3.2. Lymph Node Ratios and Other N2 Metastasis Parameters

ROC curves were plotted for the total number of lymph nodes involved in metastasis and the calculated LNR and NLR-N2 ratios. The AUC values were found to be 0.26 or LNR and 0.14 for LNR-N2 (Table 3).

The univariate analysis revealed that the total number of lymph nodes involved in a metastasis (*p* = 0.01) was a significant factor influencing survival. The optimal cut-off value for all metastatic lymph nodes was three nodes: ROC AUC 0.688 (95% CI: 0.586–0.790, *p* = 0.01). The other factors influencing survival were LNR (*p* = 0.001), with a cut-off of 0.26 (ROC AUC 0.742 [95% CI: 0.639–0.844, *p* = 0.001]), and NLR-N2 (*p* = 0.000), with a cut-off of 0.14 (ROC AUC 0.734 [95% CI: 0.655–0.813, *p* = 0.000]). In addition, the presence of single- or multistage metastases (*p* = 0.002) also significantly influenced survival; no significant influence was observed for skip metastases (*p* = 0.33). Detailed data are presented in Table 4. 

**Figure 2:**
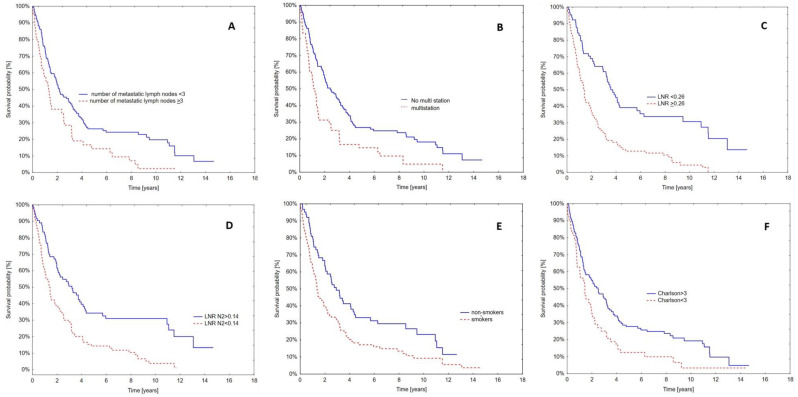
Kaplan–Meier survival curves of patients included in the study. (**A**) Number of metastatic lymph nodes; (**B**) single-/multistation; (**C**) LNR; (**D**) LNR N2; (**E**) smoking history; (**F**) Charlson Index.

The Cox-PHR model including patient clinical data showed that LNR > 0.26 (*p* = 0.004), nicotine dependence (*p* = 0.013), and CCI > 3 (*p* = 0.014) are independent prognostic factors for the survival of patients operated on due to NSCLC (Table 5, Figure 2).

## 4. Discussion

The number of metastatic lymph nodes sampled during surgery plays a significant role in the TNM classifications of malignant esophageal, gastric, colorectal, and rectal cancers [10]. Although LNR ratio has previously been studied in lung cancer [11], its prognostic value was unknown. A meta-analysis of patients with NSCLC by Zhou et al. found low LNR to be associated with significantly longer overall survival, longer disease-free survival and cancer-specific survival; in addition, patient survival was not influenced by the type of lymphadenectomy or the location of positive lymph nodes [10]. 

In our present analysis, the univariate model showed that the total number of involved lymph nodes and their nature (viz. single- or multistation) were significant prognostic factors. Similarly, previous studies indicate that the involvement of more than three N2 lymph node groups [12] and the number and location of metastases can also be important prognostic factors in NSCLC [13]. 

The present study included two nodal ratios: LNR, which considers the number of all lymph nodes involved in metastases (categories N1 and N2), and LNR-N2, which refers only to the total number of lymph mediastinal node stations (stage N2). Of the two, our univariate analysis found both to correlate with patient survival time, although LNR-N2 had a closer correlation. This is in line with a 2020 study on a group of 6245 patients, which found it to be associated with survival time in patients with NSCLC stages T1-4N1-3M0 [4]. 

However, the multivariate model found LNR to have greater prognostic value than LNR-N2 in patients operated on due to NSCLC. More specifically, LNR > 0.26, nicotine dependence, and CCI > 3 were all found to be unfavorable prognostic factors. Similarly, a previous study found LNR > 0.22 to be a significant prognostic factor for NSCLC patients [14]. Elsewhere, Han et al. found LNR > 0.36, age, sex, tumor size (T stage), and tumor differentiation to be unfavorable prognostic factors [15], while Zhang et al. found LNR < 0.19 and T feature (<3 cm) to predict favorable five-year survival among patients with NSCLC [16]. 

The prognostic value of LNR in the surgical treatment of lung cancer is doubtless influenced by the choice of open lymphadenectomy or minimally invasive lymphadenectomy. While no advantage has been found for the open technique over minimally invasive lymphadenectomy [17], the success of lymphadenectomy depends on whether the whole lymph node en bloc or only a fragment is included in the analysis [17,18]. It therefore seems necessary to first define the concept of a good lymphadenectomy. It has also been argued that the debate regarding the effectiveness of lymphadenectomy should focus less on comparing open or minimally invasive techniques and more on the question of surgical and non-surgical treatment [19].

Our work has certain limitations. Firstly, the study was single-center and retrospective in nature. Secondly, as patients were characterized by postoperative incidental N2 stage, it was not possible to avoid case selection. Finally, it was not possible to obtain complete molecular profile data for the studied patients as adjuvant treatment was provided in a different medical center. 

## 5. Conclusions

LNR and LNR N2 ratios are important prognostic factors in patients with postoperative N2 in NSCLC. Also, nicotine dependence and the presence of comorbidities with CCI > 3 appear to have predictive value and should also be considered alongside LNR index when making a prognosis. 

## Figures and Tables

**Table 1:** Patient characteristics.

Parameter	*n*	%
Sex		
F	61	37
M	102	63
Nicotinism		
Yes	100	61
No	63	39
Charlson Comorbidity Index (CCI)		
2	34	21
3	81	50
4	41	25
5	7	4
Localization—lung		
Right	76	47
Left	87	53
Localization—lobe		
left upper	49	29
left lower	37	23
right upper	37	23
middle	2	1
right lower	38	23
Surgical treatment		
Lobectomy	102	63
Bilobetomy	18	11
Pneumonectomy	43	26

**Table 2:** Pathology, adjuvant therapy, and postoperative complications.

Parameter	*n*	%
Diagnosis		
Adenocarcinoma	78	48
Squamous cell carcinoma	70	43
Large cell carcinoma	11	7
Mixed type carcinoma	4	2
Stage of the disease		
T IIIA	111	68
T IIIB	52	32
Pathological N status		
Skip metastases	47	29
Single station N2	115	71
Multi-station N2	48	29
Adiuvant chemiotherapy	141	86
Postoperative complications		
All compications	17	10
Prolonged air leak	5	
Aatelectasis	4	
Postoperative transfusion	3	
Atrial fibrillation	3	
Bronchia fistula	2	

**Table 3:** Nodal parameters included in the study N10-12—metastatic N1-stage lymph nodes; ALL—all removed lymph nodes; ALL meta—all removed metastatic lymph nodes; LNR—lymph node ratio; LNR N2—ratio of the number of N2-stage metastatic lymph nodes to the number of all dissected lymph nodes; HR—hazard ratio.

Parameter	Median	Range	HR	95% CI	*p*-Value
N10-12	3	1–9	1.05	0.95–1.16	0.29
ALL	12	6–38	1.00	0.93–1.06	0.02
ALL meta	2	1–10	1.15	1.07–1.25	0.00
LNR	0.35	0.04–0.55	6.29	3.06–12.94	0.00
LNR N2	0.16	0.03–0.75	15.82	5.26–47.51	0.00

**Table 4:** Univariate analysis of clinical factors.

	HR	HR 95% CI Lower	HR 95% CI Upper	*p* Level
Age	1.01	0.99	1.03	0.21
Sex (M)	1.08	0.77	1.54	0.49
CCI	1.35	1.07	1.7	0.01
Stage IIIA	0.81	0.56	1.15	0.1
Stage IIIB	1.23	0.86	1.75	0.1
Lobectomy	0.89	0.61	1.3	0.69
Bilobectomy	0.81	0.45	1.48	0.69
Pneumonectomy	1.12	0.77	1.63	0.69
Non-smokers	0.59	0.42	0.85	0
Adenocarcinoma	1.04	0.25	4.25	0.07
Squamous cell	1.38	0.31	6.03	0.07
Skip metastases	0.74	0.51	1.08	0.33
Single-station	1.83	1.27	2.62	0
ALL meta	1.15	1.07	1.25	0
LNR	6.29	3.06	12.94	0
LNR-N2	15.82	5.26	47.51	0

**Table 5:** Factors influencing overall survival—multivariate analysis (Cox proportional hazard analysis) including blood biomarkers with cut-off points; goodness of fit R2 = 0.198; 95% CI—95% confidence interval. HR—hazard ratio; pH—proportional hazard.

	HR	HR 95% CI Lower	HR 95% CI Upper	*p* Level	pH Chi-Square	pH *p* Level
ALL meta	1.003	0.887	1.135	0.964	0.002	0.25
LNR ALL	5.145	1.673	15.821	0.004	8.167	0.22
CCI < 3	1.351	1.061	1.72	0.015	5.971	0.92
Smoking	0.638	0.447	0.91	0.013	6.163	0.64
Multistation metastases	0.81	0.504	1.276	0.351	0.871	0.67
Skip metastases	1.034	0.656	1.631	0.885	0.021	0.6

## Data Availability

The original contributions presented in the study are included in the article, further inquiries can be directed to the corresponding author.
